# Claudin-18.2 immunohistochemical evaluation in pancreatic cancer specimens: review and recommendations for routine testing and scoring

**DOI:** 10.1007/s00428-025-04222-2

**Published:** 2025-08-21

**Authors:** Claudio Luchini, Kristina A. Matkowskyj, Takeshi Kuwata, Teri A. Longacre, Peter Schirmacher, Manabu Takamatsu, Josef Rüschoff, Matteo Fassan

**Affiliations:** 1 Department of Diagnostics and Public Health, Section of Pathology, University of Verona, Piazzale Scuro, 10, 37134 Verona, Italy; 2 ARC-Net Research Center, University of Verona, Piazzale Scuro, 10, 37134 Verona, Italy; 3 Department of Laboratory Medicine and Pathology, Mayo Clinic, Rochester, MN USA; 4 Department of Genetic Medicine and Services, National Cancer Center Hospital East, Chiba, Japan; 5 Department of Pathology, Stanford University, Stanford, CA USA; 6 Institute of Pathology, University Hospital Heidelberg, Heidelberg, Germany; 7 Center for Personalized Medicine (ZPM), Heidelberg, Germany; 8 Department of Pathology, Cancer Institute Hospital, Japanese Foundation for Cancer Research, Tokyo, Japan; 9 Division of Pathology, Cancer Institute, Japanese Foundation for Cancer Research, Tokyo, Japan; 10 Discovery Life Sciences Biomarker Services, Kassel, Germany; 11 Department of Medicine (DIMED), Surgical Pathology Unit, University of Padua, Padua, Italy; 12 Veneto Institute of Oncology, IOV-IRCCS, 35121 Padua, Italy

**Keywords:** Pancreatic cancer, PDAC, Immunohistochemistry, Predictive biomarkers, CLDN18.2

## Abstract

The evaluation of claudin-18 (CLDN18) by immunohistochemistry (IHC) has already entered routine diagnostic activity as a predictive biomarker for patients with gastric and gastroesophageal junction adenocarcinomas. Of note, the *CLDN18* gene encodes for 2 isoforms, claudin-18.1 (CLDN18.1) and 18.2 (CLD18.2). Recent evidence has shown CLDN18.2 can be expressed in a relatively high rate of cases of pancreatic ductal adenocarcinoma (PDAC). Based on these findings, preclinical research has been conducted, and clinical trials are currently underway testing anti-CLDN18.2 targeted regimens for patients affected by locally advanced unresectable and metastatic PDAC. Notably, the therapeutic strategies with specific antibodies are directed against CLDN18.2, while the antibody for IHC recognizes both isoforms, CLDN18.1 and CLDN18.2. Since CLDN18.1 is not expressed in the stomach or in the pancreas, IHC for CLDN18 in these sites can be considered specific for the isoform CLD18.2. At this time, no specific practical testing or interpretation guidelines have been proposed in this setting. However, there are several preanalytical and analytical variables and key potential pancreas-specific pitfalls, such as the frequently diffuse and strong CLDN18.2 expression in PDAC precursors, which will likely interfere with adequate CLDN18 staining and interpretation. To overcome these issues and steer the standardization of CLDN18 evaluation within the PDAC framework, this manuscript provides practical guidance on CLDN18 testing and scoring. The adoption of a standardized approach will help align all the efforts, both in research and clinical trial settings to optimally guide the most appropriate patients for anti-CLDN18.2 targeted therapies in PDAC.

## Introduction

The portfolio of molecular biomarkers that can be used in clinical practice for targeted therapies for pancreatic cancer is still quite limited. To date, in addition to *BRCA1*/*2* gene alterations that are present in up to 10% of cases, most actionable targets are restricted to the small fraction of cases (less than 10%) that are *KRAS*-wild type [1–5]. They include microsatellite instability and specific kinase fusion genes [6–8]. Recently, some promising efforts are exploring the possibility of targeting *KRAS*, but these attempts are still in clinical trials [9–11].

In this challenging oncologic scenario, claudin-18.2 (CLDN18.2) is a biomarker gaining growing attention and interest due to its frequent expression on tumor cells in a variable proportion of gastric and pancreatic cancers, among others [12–16]. Along this line, CLDN18.2 was suggested as a marker of gastric and pancreatic origin in adenocarcinomas from unknown primaries [17]. CLDN18.2 is a tight junction protein, and it is usually expressed on cell membranes with the highest prevalence in the normal gastric epithelium [9, 10]. Recently, a recombinant, chimeric, anti-CLDN18.2 monoclonal antibody called zolbetuximab has been developed against cancer cells expressing CLDN18.2 [18–21]. Notably, it has already been approved in USA, Japan, China, and Europe, for treating patients with HER2-negative and CLDN18.2-positive unresectable, advanced/recurrent gastric and gastroesophageal junction cancers [22]. It is also undergoing regulatory review with the same indications in several other countries [22].

To assess patients’ eligibility to the targeted treatment, an immunohistochemical assay for evaluating the expression of CLDN18 in cancer cells has been developed as a companion diagnostic with a monoclonal antibody (43-14A clone, Roche, Ventana) [23, 24]. This antibody recognizes both isoforms, CLDN18.1 and CLDN18.2. However, since CLDN18.1 seems to be not expressed (or expressed at a negligible level) neither in the stomach nor in the pancreas, immunohistochemistry (IHC) for CLDN18 in these sites can be considered specific for the isoform CLD18.2 [25, 26]. The modality of CLDN18 immunohistochemical testing has been recently codified in a recommendation paper for gastric cancer [23]. Of note, although significant differences and tissue-related suggestions exist between gastric and pancreatic cancer specimens, no specific recommendations on CLDN18 expression evaluation and assessment have been proposed to date for this tumor type.

To address this issue, an international panel of pathologists (C.L., T.K., T.A.L., K.A.M., P.S., M.T., J.R., M.F.) with expertise in pancreatic pathology and/or in CLDN18.2 testing/reporting met virtually in 3 half-day sessions between September and December 2024 to discuss the requirements for adequate CLDN18 immunohistochemical interpretation and reporting in pancreatic cancer. A series of virtual digitalized slides obtained from 60 different cases of pancreatic cancer, including surgical resection specimens, biopsies, and cytology, were first reviewed by all pathologists independently. Then, all cases were reviewed during a joint discussion, which was instrumental not only to reach a consensus score but also to identify the most significant issues and pitfalls in this complex scenario (see the content for gastric cancer at www.CLDN182.com, which is currently being created for pancreatic cancer).

Both CLDN18 staining (43-14A clone; Roche Ventana) and the matched hematoxylin and eosin-stained digitalized slides were available for review. The results generated from these sessions are summarized in this manuscript and are proposed as consensus recommendations for evaluating and scoring CLDN18 immunohistochemical staining in pancreatic cancer specimens and we describe our experience and our recommendations below.

## State-of-the-art review of claudin-18.2 with a focus on pancreatic cancer

### What is CLDN18.2?

The *CLDN18* gene, which is located on chromosome 3q22, encodes for the homonymous protein CLDN18 [27]. CLDN18 is a member of the class of claudins, a family of proteins first characterized in 1998 as the main components of tight junctions, the major selective barrier of the paracellular pathway between epithelial cells [28, 29]. As such, the claudins can generate the tight junction physiological barrier and control different physiological processes [28, 29]. CLDN18 is a protein comprising five domains, one cytoplasmic and four transmembrane, and two extracellular loops [27, 30, 31]. The first exon of *CLDN18* gene can be spliced in two different modalities, leading to two different splicing isoforms: CLDN18.1 and CLDN18.2. CLDN18.1 is mainly expressed by normal lung epithelium, while CLDN18.2 is diffusely expressed by the normal gastric epithelium, with the exception of the stem cell zone [31, 32].

### Is CLDN18.2 a novel therapeutic target?

The first study on CLDN18.2 with translational implications was provided in 2008 by a research investigation, where CLDN18.2 was indicated as a potential pan-cancer target suitable for therapeutic antibodies [31]. Of note, in normal epithelia, CLDN18.2 is restricted to tight junction complexes between cells, thus with limited access of its epitopes to intravenous antibodies. Conversely, upon malignant transformation, perturbations in cell polarity may lead to membrane exposure of CLDN18.2 epitopes, making them highly selective and stably expressed. Consequently, they are more accessible for targeting. The first evidence of this biological mechanism, a critical basis for therapeutic interventions, was derived from studies on CLDN18.2 expression in gastric cancer [27, 32].

Along this line, zolbetuximab (VYLOY; Astellas Pharma, initially named claudiximab or IMAB362) is a chimeric immunoglobulin G1 monoclonal antibody with high specificity and affinity to CLDN18.2. Zolbetuximab can mediate the cell death of CLDN18.2-positive cancer cells by causing antibody-dependent cellular cytotoxicity and complement-dependent cytotoxicity [23, 33]. In a first phase I clinical trial on gastric cancer, zolbetuximab monotherapy showed a manageable safety profile, tolerability, and signs of activity in patients with tumor cells with any immunohistochemical staining regardless of the percentage of positive tumor cells and staining intensity [33]. Two more recent phase III registrational randomized trials, namely SPOTLIGHT and GLOW, demonstrated improved survival indices for patients with gastric and gastroesophageal junction cancers treated with zolbetuximab in combination with chemotherapy. Specifically, regarding immunohistochemistry, inclusion criteria were HER2-negative and CLDN18.2-positive tumors, where the threshold for considering a case as positive for CLDN18.2 was set at 75% of tumor cells demonstrating moderate to high membranous staining [18, 20].

Notably, the landscape of treatment opportunities for patients with CLDN18.2-positive tumors is evolving quickly, both with the introduction of novel therapeutic regimens, including synergistic approaches of zolbetuximab with immunotherapy [34, 35] and with testing the clinical benefit derived from the administration of CLDN18.2-based targeted therapies to patients with other tumor types, starting with pancreatic cancer [36].

### What about CLDN18.2 expression in pancreatic cancer?

Recent evidence pointed out that CLDN18.2 is also expressed by pancreatic cancer tissue with a variable prevalence. A recent study based on a cohort of 130 surgically resected patients with pancreatic ductal adenocarcinoma (PDAC) showed a prevalence of 31.5% CLDN18-positive tumors and the association of positive cases with well-differentiated histology [37]. Notably, since CLDN18.1 is not expressed (or expressed at a negligible level) by pancreatic cancer cells, these results can be interpreted as specific to CLDN18.2 [25, 26]. No significant differences in terms of survival were noted between positive vs. negative cases [37]. Another study based on a similar sample size (94 cases of PDAC) and using the same threshold of previous clinical trials for classifying the cases as positive vs. negative [18, 20] confirmed a very similar prevalence (30.4%) of CLDN18-positive tumors. Furthermore, PDAC positive for CLDN18 was associated with better histological differentiation and improved survival indices compared to negative cases [38]. Improved survival indices for CLDN18-positive PDAC were also noted in another study, but the modalities of the CLDN18 scoring system were not standardized [39]. When using the threshold established for gastric cancer (≥ 75% of tumor cells expressing 2 +/3 + staining), the prevalence of CLDN18 positivity in PDAC is around 30% globally. Regarding the association of CLDN18 expression with survival, current evidence is still insufficient to support a clear survival advantage for patients with CLDN18-positive tumors.

While examining the expression of CLDN18 in the pancreas, it is of interest to report the results of its evaluation in PDAC precursor lesions, including pancreatic intraepithelial neoplasia (PanINs), intraductal papillary mucinous neoplasms (IPMNs), and mucinous cystic neoplasms (MCNs). Of note, the vast majority of such lesions, i.e., > 90% of PanINs and IPMNs and 80% of MCN, showed CLDN18 positivity [40]. This finding is not surprising, since nearly all these precursors share some degree of gastric lineage differentiation. Indeed, at the histological level, some of them (PanINs, gastric IPMN, MCNs) can show morphological foveolar- or pyloric-like epithelium, and at the immunohistochemical level, all can express gastric mucins, such as MUC5AC and MUC6 [41–43]. With that said, normal pancreatic ducts and ductal metaplasia of acinar cells were not immunoreactive for CLDN18 [39]. Since this previously referenced study was performed in 2011, no standardized approaches for immunohistochemical scoring have been adopted. Given the high rate of immunohistochemical positivity in PDAC precursor lesions, special attention of the evaluation of pancreatic specimens must be made to differentiate precursor lesions from invasive adenocarcinoma. This task may be particularly challenging in small biopsies.

A novel finding of targeting CLDN18.2 in PDAC comes as its potential role in the tumor microenvironment. In the setting of PDAC, CLDN18.2 seems to play a role in orchestrating T cell infiltration and shaping the tumor immune contexture, opening new horizons for synergistic approaches of CLDN18.2 targeting with immunotherapy in this tumor type [44].

With all these interesting observations, the possibility of adopting zolbetuximab in clinical practice for CLDN18.2 expressing PDAC is being explored in clinical trials. Along this line, the effectiveness of zolbetuximab in conjunction with chemotherapy with nab-paclitaxel and gemcitabine is currently being investigated in patients with metastatic PDAC in a phase 2 randomized clinical trial using the same CLDN18.2 scoring criteria for gastric cancer (NCT03816163; https://clinicaltrials.gov/study/NCT03816163).

### How is CLDN18.2 currently tested in pathology?

To approach the evaluation of the expression of CLDN18.2 in PDAC, it is critical to translate the lessons learned from its evaluation in gastric cancer. As an already well-established biomarker, CLDN18 expression is currently evaluated on neoplastic cells by immunohistochemistry. To date, different immunohistochemical assays are commercially available [23, 45]. One of the most critical challenges in this scenario is to provide robust evidence on the reliability of different clones and platforms and to follow standardized procedures for reducing potential biases in interpretation and reporting.

The first anti-CLDN18.2 antibody was a polyclonal antibody developed by Zymed and used for investigating CLDN18.2 expression in a recent clinical trial on gastroesophageal cancer [46]. Later on, Ganymed Pharmaceuticals AG (subsequently acquired by Astellas Pharma, Inc.) produced the CLAUDETECT 18.2 class 1 in vitro diagnostic assay. Based on its proprietary 43-14A clone, it was adopted in a subsequent clinical trial on the same tumors [47]. In collaboration with Ventana Medical Systems, Inc., the Ventana CLDN18 (clone 43-14A) assay was developed for formalin-fixed, paraffin-embedded neoplastic tissue and is now commercially available for clinical practice as a companion diagnostic assay [23]. This immunohistochemical assay is not specific for the isoform 18.2 of claudin; isoform 18.1 is typically present in the lung and not in gastric and pancreatic tissues [25, 26, 31], rendering this antibody specific for its aim. Along this line, another clone (EPR19202, Abcam) is specific for the 18.2 isoform and has shown similar results in detecting CLDN18.2-positive tumors [48].

**Fig. 1:**
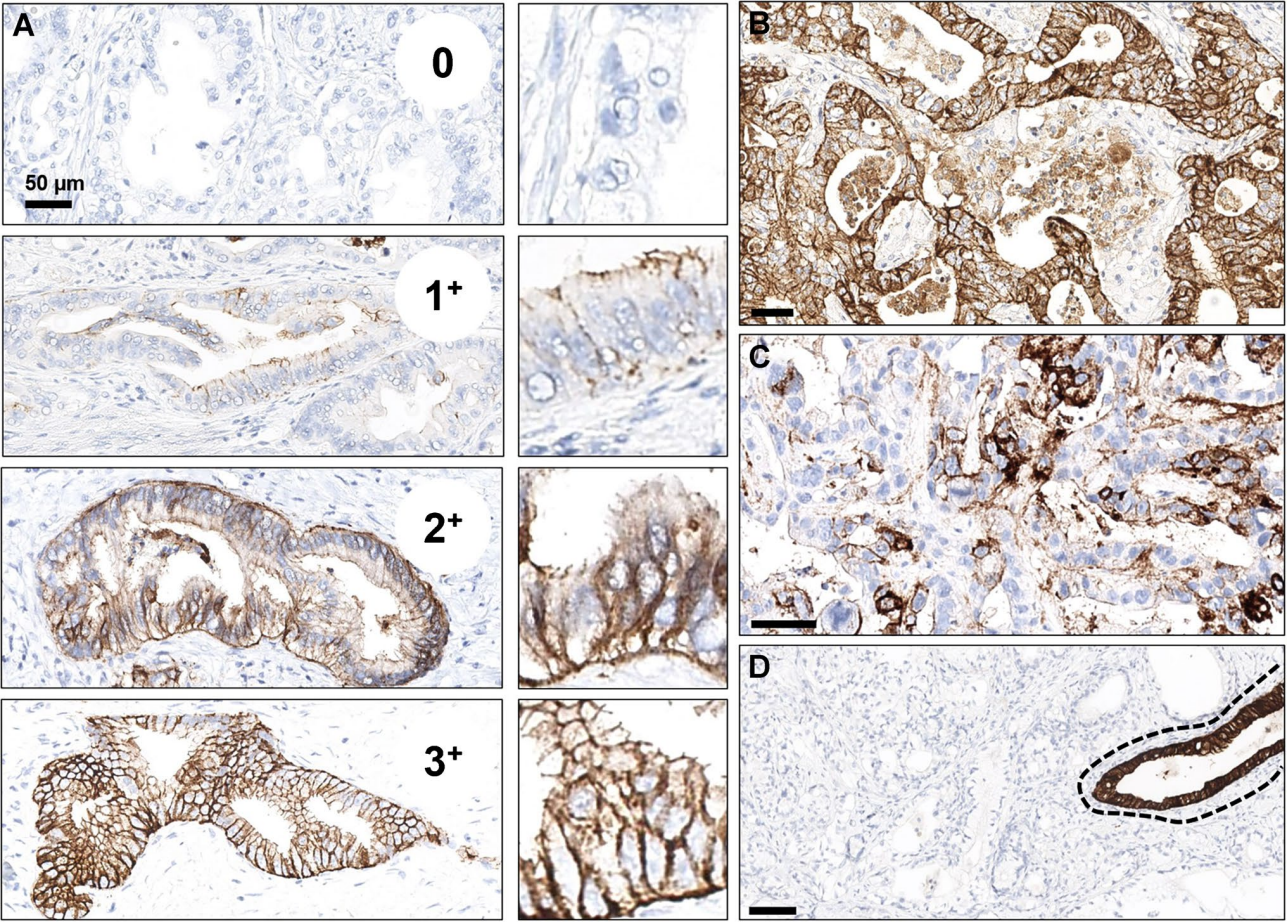
Representative figure of the different patterns of expression of Claudin 18 in pancreatic cancer. **A** Scores 0, 1 +, 2 +, and 3 + (from top to bottom and with a small central box at high 40 × obj. magnification); **B** homogeneous positivity; **C** heterogeneous diffuse positivity; **D** heterogeneous clonal positivity (within dotted line)

Given that the same claudin isoform, CLDN18.2, is expressed in both gastric and pancreatic tissues, the lessons learned from using IHC in gastric tumors could also be applicable to pancreatic tumors with a reliable biological basis. This approach can be supported only after considering the necessary distinctions based on the intrinsic properties of the different tissues, i.e., gastric and pancreatic epithelia. As was stated for gastric tumors, in PDAC, a cancer cell should also be considered claudin-positive when there is crisp membranous staining that is complete, basolateral, or lateral [23, 31, 32]. At the same time, for scoring purposes, membrane immunoreactivity with a granular pattern and/or nuclear and cytoplasmic staining should be interpreted as negative. As stated for gastric tumors [23, 49], staining intensity can also be scored in pancreatic specimens between 0 and 3 + (absent, 0; weak, 1 +; moderate, 2 +; and strong, 3 +), based on the magnification required for detecting the immunoreactivity, as follows: (1) 3 + positivity is defined as a strong brown immunoreactivity with an evident chicken-wire pattern at low-power (≤ 5 × objective/≤ 50 × magnification); (2) 2 + membranous staining is visible using a 10 × objective (100 × magnification), with 20 × useful in selected cases for confirmation of specific membrane immunoreactivity; (3) 1 + positivity can be visible using a 20 × objective (200 × magnification) but needs a 40 × objective (400 × magnification) for definitive confirmation; (4) 0: there is no membrane immunoreactivity (Fig. 1).

Of note, another methodology for CLDN18 scoring, the H-score, has been proposed for gastric tumors and adopted in a few studies on PDAC [50]. The H-score is based on the evaluation of staining intensity (weak, moderate/intermediate, and strong) similar to the previously described scores but has a different modality for its calculation. Indeed, the H-score is obtained according to the following formula: (0 × percentage of no reactive cells) + (1 × percentage of weakly stained cells) + (2 × percentage of intermediately stained cells) + (3 × percentage of strongly stained cells). Thus, based on the modality of its assessment, the H-score ranges from 0 to a maximum of 300. In PDAC, it has been proposed that a sample was considered positive with an H-score ≥ 5 [49].

Currently, the most accredited modality for identifying CLDN18 positive vs. negative cases in gastric cancer is based on calculating the percentage of 2 +/3 + positive tumor cells, considering positive those cases with ≥ 75% of 2 +/3 + cells [23]. As already stated, the biological significance of this modality of scoring is supported by the SPOTLIGHT and GLOW trials [18, 20]. However, there is no definitively established method for PDAC. For adopting a methodology in clinical practice, the expert panel highlights that it is advisable to wait for a clinical trial with positive results and use the same modality for CLDN18.2 scoring as was reported in the trial (similar algorithm to what was done for gastric cancer). Currently, we cannot make clinical recommendations regarding CLDN18 expression in PDAC. Until clinical trial data is released for pancreatic cancer, pathologists should report CLDN18 scoring following both modalities, thus reporting separately the percentage of 0, 1 +, 2 +, and 3 + cells and also the H-score. The experience with gastric tumors has shown that the first method (and in particular reporting the actual % of 2 +/3 + positive cells) seems to be more reproducible and with clinical significance, but further evidence for PDAC is needed for choosing the best methodology of CLDN18 scoring and for establishing the threshold for considering a case positive or negative. Currently, such recommendations should be taken with caution, since they may change according to upcoming results of ongoing clinical trials. Clinical/diagnostic routine recommendations would require a positive trial (optimally powered in regard to the biomarker test and biomarker optimization), a respective approval including the biomarker testing to be reflected, harmonization analyses, and at best even results of a diagnostic round robin.

## How to approach CLDN18 immunohistochemical evaluation in pancreatic cancer

### The first approach with pancreatic-specific considerations

**Fig. 2:**
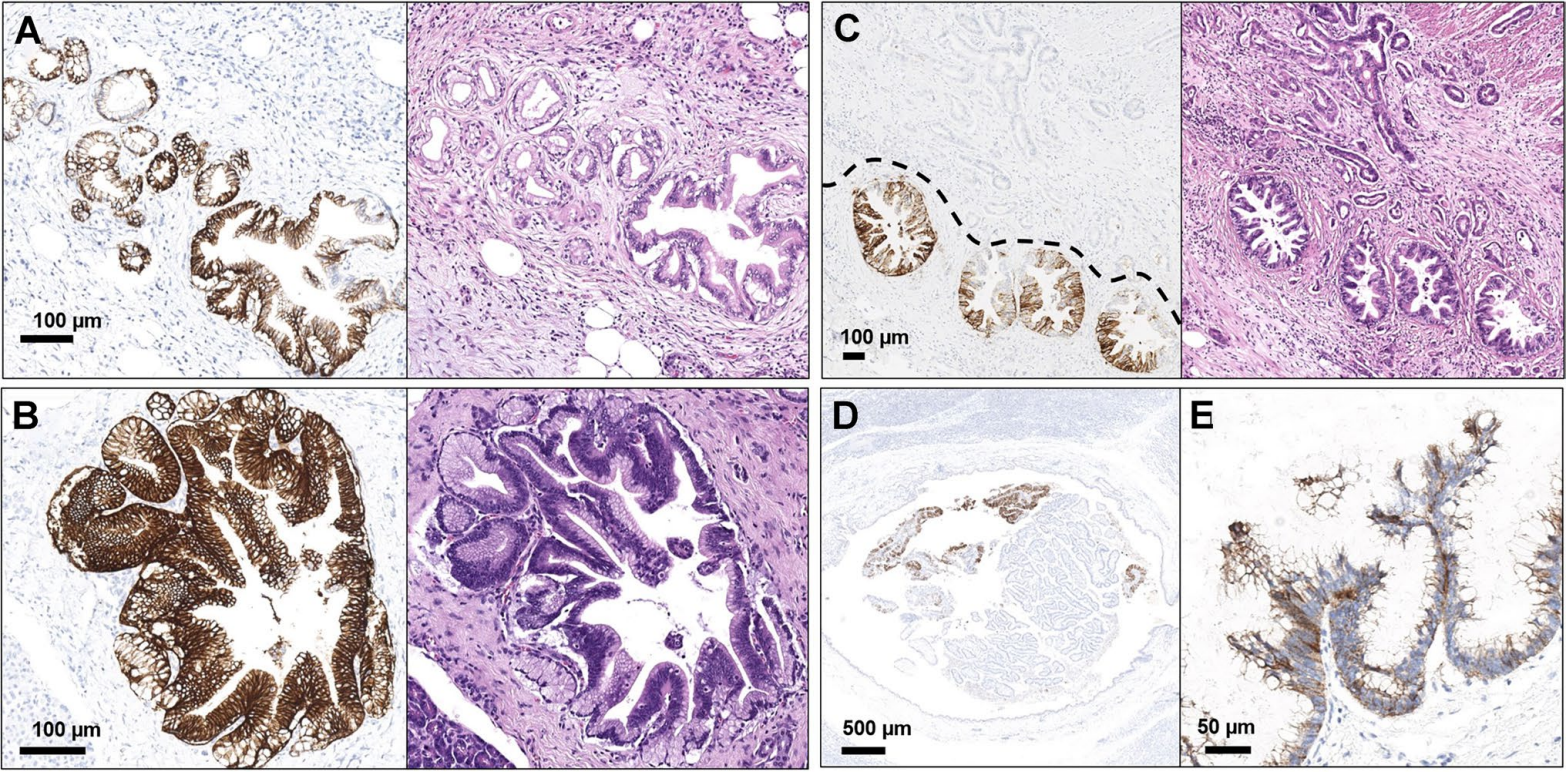
Claudin 18 positivity in pancreatic cancer precursor lesions. **A**, **B** Low-grade pancreatic intraepithelial neoplasia (PanIN); **C** high-grade PanIN (note PanIN positivity to Claudin 18 in the lower part of the image, whereas invasive cancer is negative) (**A**, **B**, **C** are provided with the corresponding hematoxylin–eosin); **D**, **E** focal Claudin 18 positivity in a case of intraductal papillary mucinous neoplasm (IPMN)

**Fig. 3:**
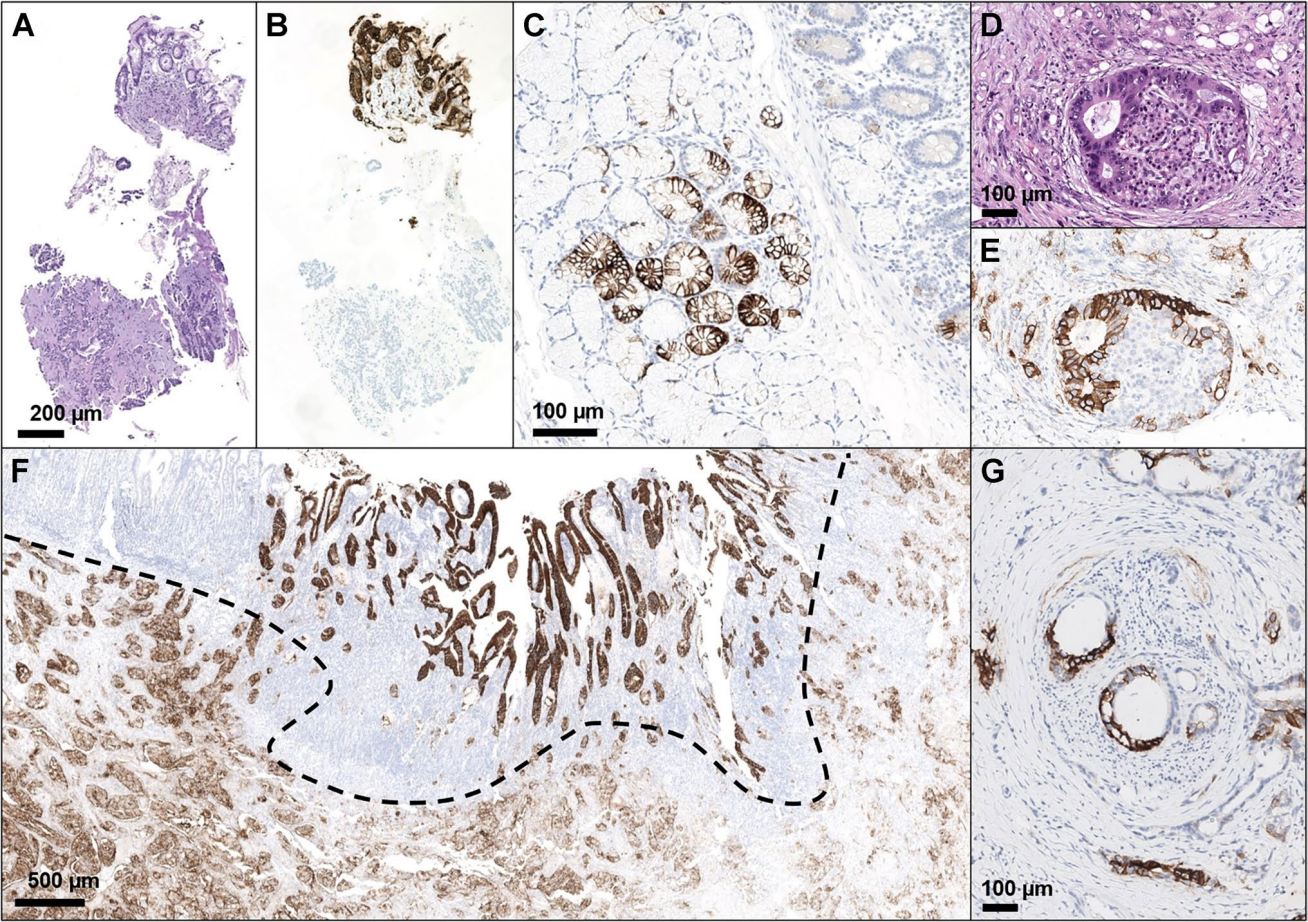
Internal positive and negative control for Claudin 18 in pancreatic specimens. **A**, **B** Peri-ampullary biopsy of pancreatic cancer infiltrating the duodenum: the fragment in the upper part contains gastric mucosa and is diffusely positive for Claudin 18, whereas the cancer (lower part) is negative. **C** Brunner’s glands are focally positive for Claudin 18; **D**, **E** Langerhans’ islets are negative for Claudin 18, even if infiltrated by pancreatic cancer, as in this case; **F** case of pancreatic cancer diffusely positive for Claudin 18 (lower part) and infiltrating the duodenum, which shows focal and strong positivity (note the strong positivity of duodenal epithelium, due to local reactive metaplasia, whereas the intramucosal glands with moderate positivity reflect direct infiltration of pancreatic cancer); **G** nerves are negative for Claudin 18, even if infiltrated by pancreatic cancer, as in this case

The initial approach to evaluate CLDN18 in pancreatic samples should start with a low magnification review (range: 2 × to 5 × objective) to obtain a general impression of the staining distribution and its heterogeneity. After this step, pathologists should focus on identifying infiltrating tumor cells and the potential presence of an internal positive control. Unlike gastric tissue, there is no normal epithelium in pancreatic samples showing immunoreactivity for CLDN18, including both ductal and acinar cells. However, some specific histopathological aspects can be used as an internal positive control in most pancreatic resection specimens. The most common one is PanINs (low and high grade). CLDN18 usually shows a 3 + immunoreactivity in these structures, most likely due to the gastric differentiation of these lesions (Fig. 2) [39, 51, 52]. Since PanINs are very common in the pancreas [53], it is often quite easy to find one of them in surgically resected specimens. Other lesions that are usually CLDN18 positive and may be found in pancreatic resection specimens include IPMNs, MCNs (Fig. 2) [40], and Brunner’s glands (focally positive; Whipple’s resections) (Fig. 3). Again, these serve as an internal positive control and are not used toward the scoring component of the tumor.

### Sample selection, management, and immunohistochemical staining

The pattern of CLDN18 expression may be heterogeneous, as has been observed in a proportion of gastric tumors. For immunohistochemical analysis in surgically resected pancreatic specimens, the tissue section area representing the entire morphological spectrum of a given PDAC should be selected for CLDN18 testing. It is well-established that PDAC can be histologically very heterogeneous with up to 10 different morphological subtypes, patterns, and clones in a single tumor [54–56]. Thus, since each of them could show different levels of protein expression, including CLDN18, all these different morphological patterns should be considered.

As in keeping with other biomarkers, IHC for CLDN18 in clinical practice should follow well-established and standardized procedures for routinely processed, formalin-fixed, paraffin-embedded tissues, according to the guidelines of the College of American Pathologists Preanalytics for Precision Medicine Project Team [57]. For surgically resected specimens, the tissue sections should be cut at approximately 4 µm and should be mounted on positively charged slides. Although antigen stability is usually maintained for at least 45 days, to preserve antigenicity, a general best practice is to cut the sections from the tissue block immediately before testing, which is also suggested for gastric cancer [23]. It is important to completely remove paraffin wax during the start of the staining process as issues in this critical step can significantly impact the specificity of the immunoreaction that create a non-specific background stain.

### An insight on positive and negative controls

In routine evaluation of immunohistochemical analysis, positive and negative controls play a crucial role as fundamental support of the reliability of the results. As already indicated, internal positive controls in pancreatic specimens can be represented by PanINs and by other less common entities, including macroscopic precursor lesions (e.g., IPMNs, MCNs) and normal structures such as Brunner’s glands (focally positive) (Figs. 2 and 3). Internal negative controls, which must be completely CLDN18 negative, are normal pancreatic ducts and acinar cells, Langerhans’s islets, stromal cells (including tumor-associated desmoplastic reaction), inflammatory cells, intra- and extra-parenchymal adipocytes, blood vessels, and peripheral nerves (Fig. 3).

Based on these considerations, pathologists should select for CLDN18 testing not only PDAC, but at least one negative and one positive control. This task may be challenging or even impossible in the case of CLDN18 testing in non-surgical specimens, where PanINs and other positive controls can be lacking. To overcome this issue, non-neoplastic gastric tissue from a test sample can serve as positive run control; it can be put on the same slide for IHC (on-slide control).

### Can the sample type impact CLDN18 testing results? And what about cytology?

For all immunohistochemical testing, surgically resected specimens represent the gold standard in terms of material for analysis. They offer different advantages: (i) internal positive and negative controls are easier to identify and select; (ii) the evaluation of large tumor areas overcomes issues related to intratumor heterogeneity, both at morphological and CLND18 expression levels; (iii) hypo/hyper-fixation artifacts can be recognized almost immediately (e.g., shrinkage artifacts and gradient staining); (iv) unreliable reaction (failure in internal positive and negative control) or incorrect patterns (e.g., cytoplasmic immunoreactivity), and another tissue block for repeating the analysis is usually available.

**Fig. 4:**
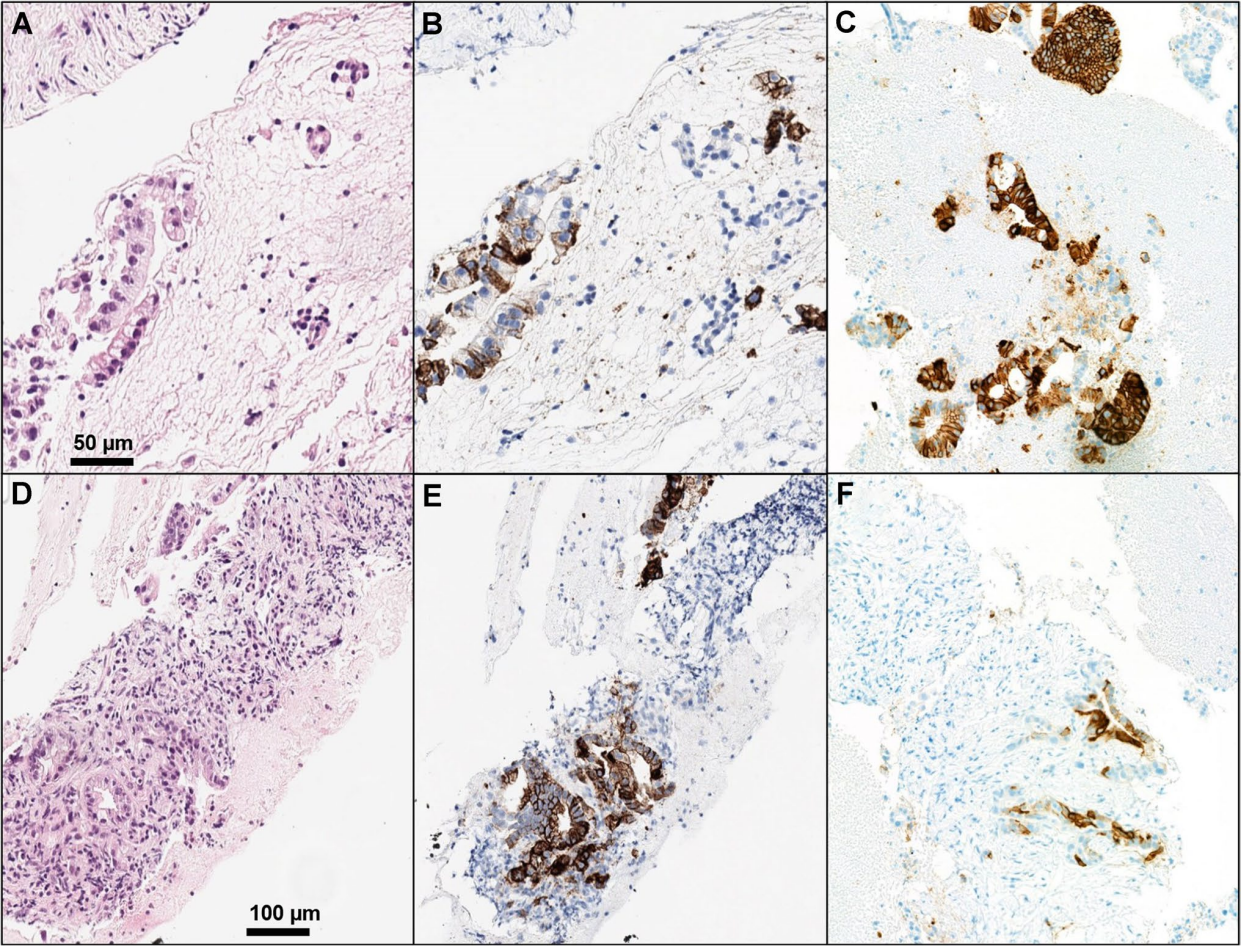
**A**, **B**, **C** Claudin 18 assessment in cytological samples is very challenging and even impossible, due to the difficulties in discriminating neoplastic cells from invasive cancer from precursor lesions (**A** and **B** show the same case). **D**, **E**, **F** Claudin 18 assessment in biopsies is challenging but it can help to avoid misinterpretation (**D**, **E** PanINs/non-invasive ducts; **F** invasive glands with Claudin 18 positivity)

Notably, it should be highlighted that most patients with PDAC have locally advanced or metastatic disease at the time of diagnosis, and thus are often not amenable to surgical approaches [58, 59]. The translation of this critical consideration in a real-world practice is that surgical resection specimens will not represent the majority of the material submitted for CLDN18 testing. Two possible types of specimens are usually collected in this setting: fine-needle aspiration cytology (FNAC) and fine-needle biopsy (FNB) [60–62]. These mini-invasive approaches represent the standard of care for diagnostic purposes [60–62]. For evaluating CLDN18 immunohistochemistry, the panel of coauthors agreed that FNB usually represents adequate material for its assessment [63]. Indeed, in FNB, it is often possible to discriminate between high-grade PanINs (typically CLDN18 positive) vs. invasive glands of PDAC, whereas in cytological specimens (cell blocks), this task can be very challenging or even impossible (Fig. 4). This is a very significant point, since this aspect may lead to false positive results in the evaluation of CLDN18 in FNAC. In any case, the use of heterologous tissue (e.g., normal gastric tissue) as on slide control appears even more important in this type of material, where internal positive control can be totally lacking. The use of cytological samples to assess CLDN18 expression should be avoided; it may be possible only in highly specialized centers and by pathologists with extensive experience in the field, but in a non-negligible fraction of cases, such evaluation will remain impossible (Fig. 4). In selected cases, IHC for SMAD4 (DPC4) in cell blocks may help identify those cells belonging to an invasive PDAC. Indeed, *SMAD4* alterations represent a late event in PDAC carcinogenesis and when present, they usually indicate the presence of an infiltrative tumor [64, 65]. Thus, SMAD4 IHC could be used in cell blocks to identify those cells where CLDN18 could be evaluated. *SMAD4* alterations usually lead to a defect in the homonymous protein production and consequently a loss of SMAD4 nuclear expression in neoplastic cells and supports the diagnosis of PDAC [62, 64, 65]. However, IHC for SMAD4 suffers from some potential biases, including a high vulnerability to non-optimal pre-analytical conditions and weak patterns of immunoreactivity that may be difficult to interpret [62]. Furthermore, a significant fraction (around 45–50%) of PDACs does not harbor *SMAD4* alterations; consequently, in those cases, this immunohistochemical analysis is not helpful.

Given the high rate of metastases, which are present in the majority of patients affected by PDAC already at the time of the diagnosis, pathologists may be called for evaluating CLDN18 expression also on biopsies from metastatic sites. It should be acknowledged that there are few data in the literature regarding the expression of CLDN18 in PDAC metastases as well as on the fidelity of its expression between matched primary tumors and metastases. However, it seems that the positivity rate of CLDN18 expression in metastases is similar to that of pancreatic primaries, and that CLDN18 expression is similar in primary tumors and matched metastases [66]. The panel highlights that more data on this specific topic are needed for supporting more definitive conclusions.

Another consideration regards the adequacy of material, given the potential heterogeneity of CLDN18 expression. A recent study highlighted different rates of CLDN18 positivity in FNBs vs. surgical specimens [58]; this striking finding leads the consensus panel to its strong recommendation that at least 50 viable infiltrating tumor cells should be evaluated in order to obtain a reliable result.

Overall, it should be also acknowledged that, regardless of the sample type, a detailed report on claudin-18.2 expression can be time consuming and difficult to integrate into normal daily workflow. This is even more evident when considering the potential pitfalls that can be encountered in clinical practice (see below). Nowadays, additional biomarker testing and assessment should be discussed in a multidisciplinary setting, with shared responsibilities on the indications and importance of that specific analysis in that particular patient.

### A focus on potential pitfalls in CLDN18.2 assessment

#### Cytoplasmic or nuclear staining

As already stated for gastric cancer [23], only tumor cells with perceptible and convincing linear immunoreactivity for CLDN18 should be considered positive. Thus, cytoplasmic or nuclear staining is not scored. It should be noted that, in some cases with a strong positivity to CLDN18, the membranous staining pattern may be substantially obscured by cytoplasmic immunoreaction, but usually, a chicken wire pattern can still be observed. In cases with ambiguous staining, as stated for gastric cancer [23], cautious interpretation is recommended. In cases with relevant artifacts associated with cytoplasmic staining (e.g., preanalytic issues, thermal effects), to reduce the risk of false-positive or false-negative results, an alternative tumor sample should be selected when available. If it is not available (e.g., FNB), a second attempt should be performed on the same material. In the case of the same results, the final pathology report is “inadequate for CLDN18 evaluation.” If the artifacts are limited to only a tumor area, the CLDN18 scoring should only be performed in the PDAC region lacking those artifacts.

#### Precursor lesions

As already stated, precursor lesions represent the major cause of potential errors and above all, false-positive, in the evaluation of CLDN18 expression in pancreatic specimens. On the one hand, the presence of PanINs, IPMNs, and MCNs can be of relevant help in evaluating the reliability of immunohistochemistry, serving as internal positive controls. On the other hand, they can generate staining patterns of challenging interpretation; their positivity should be excluded from CLDN18 scoring.

#### Aberrant positivity

Cases showing aberrant weak cytoplasmic immunoreactivity in inflammatory cells (e.g., macrophages) or other non-neoplastic elements have been rarely observed and should not be scored. Typically, such cases showed apparent preanalytical associated issues. Other patterns of aberrant positivity can be rarely seen in IPMN-associated pools of mucin or within necrotic intraglandular debris. All aberrant immunoreactions should be excluded from CLDN18 scoring.

#### Preanalytical artifacts

IHC typically suffers from errors derived from the preanalytical phase. In evaluating pancreatic specimens subjected to electrocautery (especially the retroperitoneal margin), they can appear histologically torn and coagulated. Maintenance of antigenicity may be affected in such samples, and all PDAC regions showing thermal artifacts should be excluded from CLDN18 scoring. Under-fixation may also damage antigen preservation and cause false-negative staining along with edge effect and nonspecific cytoplasmic staining, similar to what has been observed for gastric samples [23].

### Ideal CLDN18.2 pathology report

**Fig. 5:**
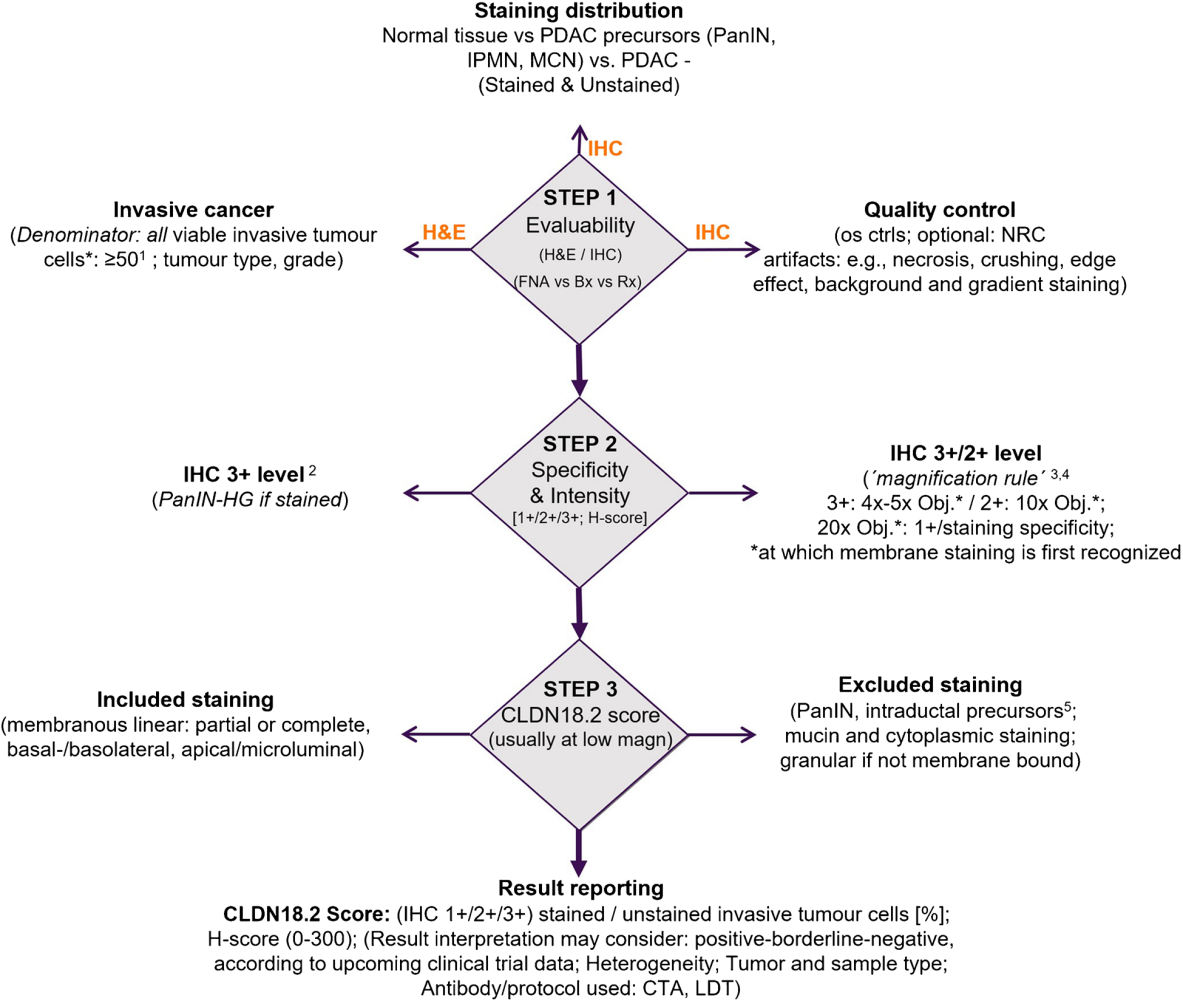
Diagnostic algorithm with a stepwise approach for the assessment of Claudin 18 in pancreatic specimens. Abbreviations in the figure: os ctrls, on-slide controls; PanIN, pancreatic intraepithelial neoplasia; IPMN, intraductal papillary mucinous neoplasms; MCN, mucinous cystic neoplasms; HG, high grade of dysplasia; Bx, biopsies; CTA, clinical trial application; FNA, fine needle aspirates; H&E, hemtoxylin and eosin; IHC, immunohistochemistry; LDT, lab developed test; NRC, negative reagent control; Obj, microscope objective; Rx, resection. ^1^Ventana 43-14A Assay manual; ^2^Schildhaus HU et al. Clin Cancer Res. 21:907–15, 2015; ^3^Scheel AH et al. Diagn Pathol. 13(1):19. 2018. ^4^Fassan M et al. Mod Pathol. 37:100,589, 2024; ^5^IPMN and MCN are considered macroscopic precursor lesions of pancreatic cancer

Standardizing pathology reports, including those reporting biomarkers and their interpretation, is crucial for addressing the best therapeutic strategies in precision oncology. All findings derived from the workflow in the pathology laboratory should be presented with a clear and concise modality to rapidly transmit all the information to the oncologists (with common sense). Along this line, the ideal CLDN18 expression report should follow a precise algorithm (Fig. 5) and contain the following information:

- Type of specimen used for analysis (surgical resection specimen/FNB/FNAC/other)
- Site of sampling (it is important to guide the correct interpretation of the eventual presence of positive but not clinically relevant tissue, such as PanINs, macroscopic precursor lesions, and also normal gastric epithelium derived from EUS-guided FNB/FNAC)
- Sample adequacy, and if inadequate, the causes of inadequacy should be explicitly stated (e.g., lack of tumor cells, electrocautery/fixation artifacts)
- Antibody clone and immunohistochemical stainer used for the testing
- Companion diagnostic or in-house-developed test
- Test results as the percentage of membranous 1 +, 2 +, and 3 + positive cells, assessed separately
- Additional analytical and clinical interpretative comments (e.g., qualification of the laboratory and bibliographical references), if needed.

## Conclusions

CLDN18 expression tested by IHC has already entered daily clinical practice for selecting patients with locally advanced and metastatic gastric and gastroesophageal junction adenocarcinomas for anti-CLDN18.2-targeted therapy [22]. The evidence regarding CLDN18 expression in pancreatic tissues and PDAC paves promising horizons towards adopting similar approaches in the near future for patients affected by pancreatic cancer, pending the results of ongoing clinical trials. Following this perspective, we have attempted to provide a general guide for CLDN18 scoring in pancreatic specimens. This manuscript, also based on the evaluation and consensus/discussion of 60 different cases, includes a specific focus with tips and tricks for staining, interpretation, and potential pitfalls. A standardized modality for CLDN18 scoring is crucial for research studies in the field and, hopefully, may become part of the standard of care for patients affected by PDAC. As pathologists play a crucial role in the correct interpretation of predictive biomarkers for precision oncology [67]

## Data Availability

All data/information are available upon request to the corresponding author.
